# Exceptional points enhance sensing in silicon micromechanical resonators

**DOI:** 10.1038/s41378-023-00641-w

**Published:** 2024-01-19

**Authors:** Man-Na Zhang, Lei Dong, Li-Feng Wang, Qing-An Huang

**Affiliations:** Key Laboratory of MEMS of the Ministry of Education, Southeast University, Nanjing, 210096 China

**Keywords:** Electrical and electronic engineering, Physics

## Abstract

Exceptional points (EPs) have recently emerged as a new method for engineering the response of open physical systems, that is, systems that interact with the environment. The systems at the EPs exhibit a strong response to a small perturbation. Here, we show a method by which the sensitivity of silicon resonant sensors can be enhanced when operated at EPs. In our experiments, we use a pair of mechanically coupled silicon micromechanical resonators constituting a parity–time (PT)-symmetric dimer. Small perturbations introduced on the mechanically coupled spring cause the frequency to split from the EPs into the PT-symmetric regime without broadening the two spectrum linewidths, and this frequency splitting scales with the square root of the perturbation strength. The overall signal-to-noise ratio is still greatly enhanced, although the measured noise spectral density of the EP sensing scheme has a slight increase comparable to the traditional counterpart. Our results pave the way for resonant sensors with ultrahigh sensitivity.

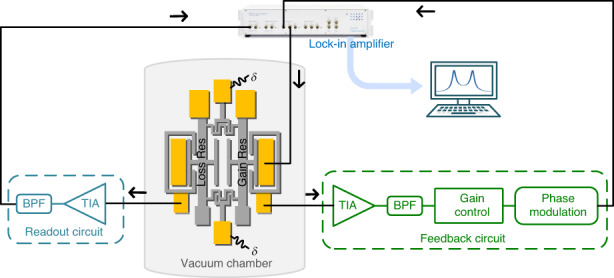

## Introduction

**Fig. 1:**
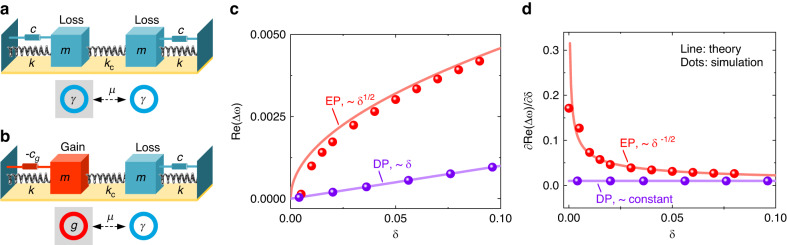
Principle of the frequency monitoring of two coupled micromechanical resonators. The two resonators with identical mass $m$ and identical stiffness $k$ are coupled with the coupling strength $\mu ={k}_{c}/k$ where ${k}_{c}$ is the coupling spring constant. **a** Traditional scheme. Two coupled resonators with the same loss $\gamma =c/\sqrt{{mk}}$ where *c* is the damping coefficient. **b** PT-symmetric dimer. One resonator with loss $\gamma$ and the other resonator with an equivalent amount of gain *g*. **c** Comparison of the frequency splitting $\Delta \omega$ of the two coupled resonators operated at the diabolic point (DP) and the exceptional point (EP) when the coupling spring is subject to an external perturbation $\delta =\Delta {k}_{c}/{k}_{c}$. The response $\Delta \omega \propto {\delta }^{1/2}$ for the EP resonators whereas $\Delta \omega \propto \delta$ for the DP resonators. **d** Comparison of the sensitivity of frequency splitting to perturbation. For small perturbations, the sensitivity of the EP resonators is enhanced by an order of magnitude with respect to that of the DP resonators. In computation, the gain/loss coefficient $g=\gamma =0.01$ and the initial coupling coefficient $\mu =0.01$ are set for the EP resonators. The line and dots in (**c**) and (**d**) indicate the theoretical and simulated results, respectively

The concept of microelectromechanical system (MEMS) resonators that mechanically vibrate at resonance has a long history of research dating back to the 1960s^1^. The resonator is often utilized for resonant sensors that generate significant development and commercial applications associated with charge, mass, displacement, acceleration, and magnetic sensing^2^. Parameters of interest to be sensed, i.e., small perturbations, induce the effective stiffness change or mass change of the resonator, leading to its resonant frequency shift or vibrating amplitude variation. Traditionally, the resonant sensor in the form of a frequency shift as an output signal has a quasidigital nature. As a result, it is basically independent of analog levels and minimizes the inaccuracies that arise in an analog output as well as its converted digital format^3,4^. However, the frequency shift is proportional only to small perturbations, leading to low sensitivity. By biasing the resonant sensor in a nonlinear state or in high-order frequency mode, the enhanced sensitivity has been explored^5,6^. Based on the mode localization effect of weakly coupled resonators, a resonant sensor in the form of an amplitude ratio as an output signal has been extensively developed^7–9^. For the often used mode-localized resonators shown in Fig. 1a, where two identical resonators of proof mass *m*, mechanical spring constant *k*, and loss strength *γ* are weakly coupled through a mechanical spring constant *k*_*c*_, a perturbation induces the effective stiffness change Δ*k* or mass change Δ*m* for one of the two resonators, resulting in amplitude variations. This class of sensors offers high sensitivity, but monitoring the voltage or current amplitude for the analog output is challenging at the same level of precision as in tracking the frequency shift.

The development of a resonant sensor that offers high sensitivity while maintaining high precision is fundamentally needed. Here, we propose a PT-symmetric scheme in which an equivalent amount of gain, controlled actively by a closed-loop feedback circuit, is incorporated into one resonator that serves as a PT-reversed counterpart to the other resonator with loss (Fig. 1b). We theoretically propose and experimentally demonstrate that the frequency splitting of PT-symmetric resonators when operated at EPs scales with the square root of the perturbation strength, in contrast to the linear frequency shift of the traditional scheme (Fig. 1c).

The PT-symmetry concept originated in the context of quantum mechanics^10,11^ and has been extensively explored in classic wave systems, such as optics and photonics^12–15^, acoustics^16,17^, mechanics^18,19^, and electronics^20,21^. PT-symmetric systems have two distinguished phases, an exact PT-symmetric phase with real eigenvalues and a broken PT-symmetric phase with complex-conjugate eigenvalues. EPs where both eigenvalues and eigenvectors coalesce separate the exact phase from the broken phase. Systems at the EPs exhibit strong responses to a small perturbations. Therefore, EP-based sensors have recently received significant attention^22–25^, although there is an ongoing debate about their fundamental limits^26–28^. In the case of PT-symmetric inductor-capacitor (LC) resonators, classic noises are more relevant than quantum noises. The enhanced sensitivity of PT-symmetric LC sensors has been experimentally demonstrated by biasing them at the exact phase^29,30^, EPs^31,32^, and broken phase^33^. Moreover, through optimizing the design of low pass circuits, thermal noises have been alleviated to an identical level as that achieved by the corresponding traditional sensing scheme^34^. Inspired by these works, in this paper, we explore the consequence of PT-symmetric silicon micromechanical resonators for EP-enhanced sensing.

## Principle of EPs-enhanced sensitivity

To describe how EPs enhance sensing in silicon micromechanical resonators, we present an analysis based on a PT-symmetric dimer^11^ consisting of two identical resonators of mass *m*, spring constant *k*, and resonance frequency ${\omega }_{0}=\sqrt{k/m}$, as shown in Fig. 1b. In the PT-symmetric dimer, one resonator has a loss $\gamma =c/\sqrt{{mk}}$, and the other has a gain $g={c}_{g}/\sqrt{{mk}}$, with *c* and *c*_*g*_ representing the damping coefficients of the loss and gain resonators, respectively. The resonators are coupled together with the coupling strength $\mu ={k}_{c}/k$, where ${k}_{c}$ is the coupling spring constant. The system is described by

1 $$\left[\begin{array}{cc}1+\mu -i\omega g-{\omega }^{2} & -\mu \\ -\mu & 1+\mu +i\omega \gamma -{\omega }^{2}\end{array}\right]\left[\begin{array}{c}{x}_{1}\\ {x}_{2}\end{array}\right]=0$$

where *ω* is the frequency scaled by ${\omega }_{0}$, the subscript 1 (or 2) refers to the gain (or loss) resonator, and ${x}_{\mathrm{1,2}}$ are the eigenstates describing displacements. In the case of weak coupling, Eq. (1) may be cast into the following coupled-mode model (see Methods):

2 $$-i\frac{d}{{dt}}\left[\begin{array}{c}{x}_{1}\\ {x}_{2}\end{array}\right]=\left[\begin{array}{cc}1+\frac{\mu -{ig}}{2} & -\frac{\mu }{2}\\ -\frac{\mu }{2} & 1+\frac{\mu +i\gamma }{2}\end{array}\right]\left[\begin{array}{c}{x}_{1}\\ {x}_{2}\end{array}\right].$$

To find the eigenfrequencies, taking ${x}_{\mathrm{1,2}}\propto {e}^{i\omega t}$, we obtain the characteristic equation

3 $$\left(1+\frac{\mu -{ig}}{2}-\omega \right)\left(1+\frac{\mu +i\gamma }{2}-\omega \right)-\frac{{\mu }^{2}}{4}=0.$$

Given a delicate balance between gain and loss, $g=\gamma$, the eigenfrequencies and the corresponding eigenstates are given by

4a $${\omega }_{\pm }=1+\frac{\mu }{2}\pm \frac{1}{2}\sqrt{{\mu }^{2}-{\gamma }^{2}}.$$

4b $${\left[\begin{array}{c}{x}_{1}\\ {x}_{2}\end{array}\right]}_{+}=\frac{i}{\sqrt{2\cos \varphi }}\left[\begin{array}{c}{{\rm{e}}}^{-i\frac{\varphi }{2}}\\ {-{\rm{e}}}^{+i\frac{\varphi }{2}}\end{array}\right]$$

4c $${\left[\begin{array}{c}{x}_{1}\\ {x}_{2}\end{array}\right]}_{-}=\frac{1}{\sqrt{2\cos \varphi }}\left[\begin{array}{c}{{\rm{e}}}^{+i\frac{\varphi }{2}}\\ {{\rm{e}}}^{-i\frac{\varphi }{2}}\end{array}\right]$$

with $\varphi ={\tan }^{-1}\frac{\gamma }{\sqrt{{\mu }^{2}-{\gamma }^{2}}}$, where $\varphi$ is the phase difference between resonator 1 and resonator 2.

**Fig. 2:**
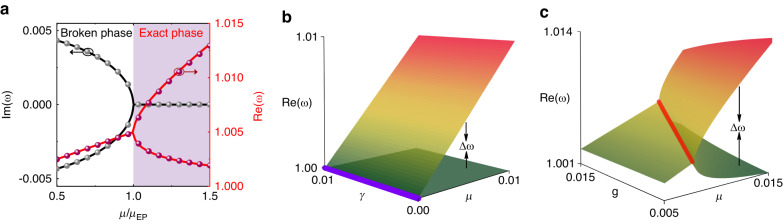
Sensitivity enhancement of silicon micromechanical resonators biased at EP. **a** The real and imaginary parts of the eigenfrequencies for the EP resonators are shown as a function of the normalized coupling coefficient $\mu /{\mu }_{{EP}}$ with $g=\gamma =0.01$. The line and dots denote the theoretical and simulated results, respectively. **b** The real frequency evolutions of the DP resonators when varying the coupling coefficient $\mu$ and the loss $\gamma$. The operation of the DP resonators is usually required to be lossless, and, hence, its frequency is independent of loss. **c** The real frequency evolutions of the EP resonators when varying the coupling coefficient $\mu$ and gain $g$ with $\gamma =0.01$. Even if $g\ne \gamma$, the real parts of the complex frequency also respond to the coupling. The magnitude of the response of the resonators is defined by the frequency splitting $\Delta \omega$

Note that the eigenfrequencies depend upon the coupling strength $\mu$ relative to the gain/loss parameter *γ*. In the exact phase $\mu > \gamma$, the coupling between the gain and loss resonators is sufficiently strong. The eigenfrequencies are real, which is characterized by equal magnitudes for the superposition oscillations on the gain and loss sides. In the broken phase $\mu < \gamma$, the coupling is too weak for the system to remain in equilibrium, and the eigenfrequencies become complex with a single real frequency and conjugate imaginary parts, which indicates that it grows exponentially in one mode and decays exponentially in the other. When $\mu =\gamma ={\mu }_{{\rm{EP}}}$, the eigenfrequencies are merged into ${\omega }_{{\rm{EP}}}=1+\mu /2$, i.e., EPs at which the eigenfrequencies and the corresponding eigenstates coalesce. Figure 2a shows the evolution of the real and imaginary parts of the eigenfrequencies with coupling strength $\mu$. When the coupling spring is subjected to an external perturbation, the coupling spring constant ${k}_{c}$ is altered to ${k}_{c}+\triangle {k}_{c}$, corresponding to the coupling strength $(1+\delta )\mu$, where $\delta =\triangle {k}_{c}/{k}_{c}$. Solving Eq. (2)∼(3) under the perturbation yields the frequency splitting near EPs, ${\triangle \omega }_{{\rm{EP}}}=\mu \sqrt{2\delta }$, and its sensitivity to perturbation, $\partial {\triangle \omega }_{{\rm{EP}}}/\partial \delta =\mu /\sqrt{2\delta }$. Physically, an external perturbation pushes the system away from the EP and consequently lifts the non-Hermitian degeneracy of the eigenfrequencies and the corresponding eigenstates, triggering frequency splitting^10,11^. In our scheme, the perturbation *δ* is positive because of the electrostatic force, which is always attractive. Therefore, the eigenfrequency and its splitting are real during operation. The perturbation in the previous EP sensing scheme causes the systems to break, giving rise to complex frequencies^22–25^. The presence of the imaginary part of the eigenfrequencies leads to broadening and further overlapping of the two adjacent spectra and sets a fundamental resolution limit on the sensitivity^27^. In fact, the perturbation in our scheme drives PT-symmetric resonators to move from the EP into PT-symmetric regimes. This indicates that silicon micromechanical resonators that operate at EPs can be exploited for enhanced sensing using frequency splitting as a measure, as shown in Fig. 2c. In contrast, traditional resonators utilize a diabolic point (DP) at which the eigenfrequencies, but not the eigenstates, coalesce, as described in the Methods section. The traditional resonators become trivially degenerate when uncoupled from each other, $\mu =0$. When coupled and subject to the same perturbation $\delta$, the resulting frequency splitting is proportional to the perturbation strength, ${\triangle \omega }_{{\rm{DP}}}=\mu \delta$, as shown in Fig. 2b. Hence, for sufficiently small perturbations, the splitting at the EP is larger than that at the DP. We use finite-element simulations to validate the above results, as provided in the Supplementary Material Fig. 1c, d (dots) show the frequency splitting and its sensitivity as a function of the perturbation for the EP and DP resonators, respectively. Figure 2a (dots) shows the real and imaginary parts of the eigenfrequencies as a function of the coupling strength. These results confirm the coupled-mode model.

## Experiments of EP-enhanced sensitivity

**Fig. 3:**
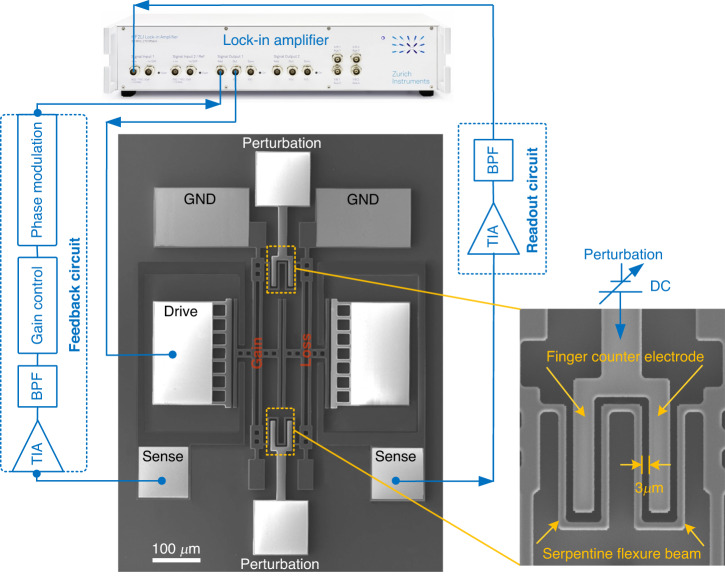
Experiments of EPs-enhanced sensing in silicon micromechanical resonators. An SEM image of two coupled DETF resonators fabricated on SOI substrates. Both resonators are connected by a serpentine flexure beam (partial enlarged drawing) for weak coupling, and the finger counter electrode is connected to the anchor. A schematic of the measurement setup and their connections are plotted. Both the AC driving signal and feedback control signal are simultaneously applied to the gain resonator. The frequency response was recorded using a lock-in amplifier connected to the loss resonator. The fabricated resonators were tested in a custom vacuum chamber

We demonstrate the theory presented above using a pair of mechanically coupled silicon micromechanical resonators. A scanning electron micrograph (SEM) image of the structure is shown in Fig. 3. Both resonators consist of double-ended tuning forks (DETFs). Previously, a pair of electrically coupled nearly identical DETFs were used to demonstrate the mode localization effect^7,8^. Our work differs in that we aim to demonstrate a scheme of EP-enhanced sensitivity. The gain resonator is regulated actively by external proportional feedback control. During external feedback, the resonator motion is transduced into a capacitance variation, leading to a current variation that is then filtered, phase shifted, and finally applied to drive the resonator^35^. Here, the DETF on the left is configured as a gain resonator, with the feedback circuit connected to its sense electrode. The DETF on the right is designed as a loss resonator, and the readout circuit is connected to its sense electrode (Supplementary Fig. 5a).

Gain is finely tuned by adjusting the amplitude of the feedback force that is in phase with the mechanical velocity so that a delicate balance between gain and loss can be achieved. As shown in Fig. 3, the two DETFs are weakly coupled by a serpentine flexure beam connected to their ends. The equivalent spring stiffness of the flexure beam can be electrostatically adjusted^36^. The counter electrode and flexure beam are directly designed to be opposite to each other with a gap of 3 μm. To demonstrate the physical phenomenon of EP-enhanced sensing in resonators, the voltage applied across them can precisely adjust the equivalent spring stiffness of the flexure beam to generate small perturbations (Supplementary Fig. 3c).

Through simulations, we fabricated a pair of mechanically coupled nearly identical DETF resonators, as shown in Fig. 3. The gain resonator was driven and sensed using parallel-plate capacitive transduction, while the loss resonator with parallel-plate capacitive transduction was constructed only for measurement. The fabricated resonators were tested under a vacuum (≈1.65 Torr) in a custom vacuum chamber. The frequency response was recorded using a lock-in amplifier connected to the loss resonator. Both the alternating current (AC) driving signal and feedback control signal were simultaneously applied to the gain resonator. A quality factor of approximately 350 was measured, and the corresponding loss$\gamma$ and damping coefficient *c* were estimated as 0.00285 and 4.77 × 10^−6^ N·s/m, respectively. Due to manufacturing process tolerances, there is a deviation between the initial frequencies of the two resonators. By adjusting the feedback amplitude, the coupled resonators were brought closer to the EPs, at which the resonance frequency was measured to be approximately 302.36 kHz. The perturbation was then applied by regulating the direct current (DC) voltage across the flexure beam and its counter electrode.

**Fig. 4:**
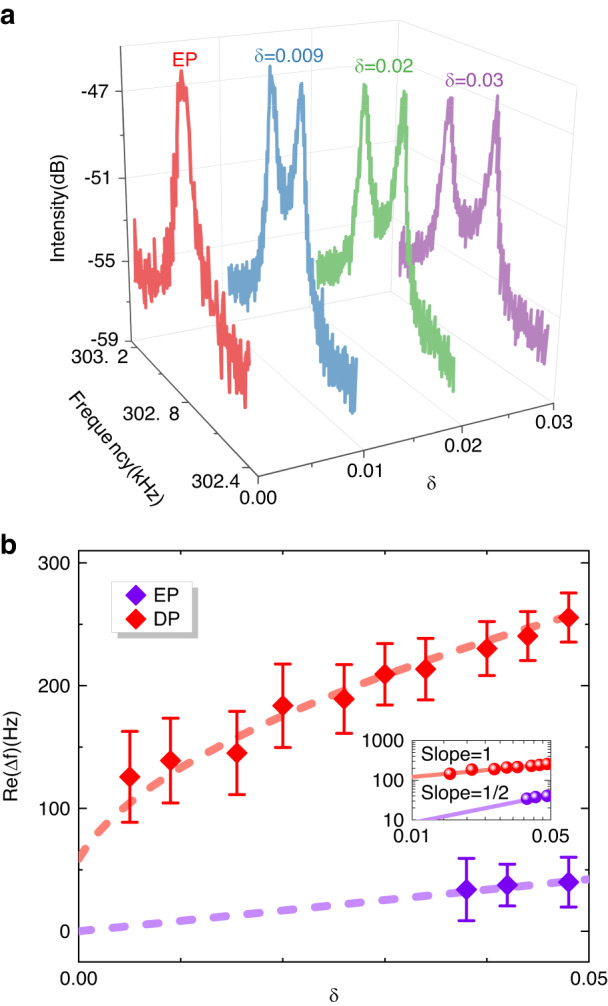
Measurements of PT-symmetric resonators operated near EPs. **a** The frequency response of the EP resonator as a function of perturbation *δ* = 0.9%, 2%, and 3%, respectively. **b** The real parts of the frequency splitting as a function of perturbation *δ* around EP (red). The dotted lines indicate the fitted square-root behavior, the filled diamond indicate experimental data, and the error bars indicate the uncertainty in frequency measurements due to the external circuits. For comparison, the results of DP resonators are shown in brown. The inset displays a logarithmic plot of the dependence of the frequency splitting on *δ*. The EP resonator exhibits a slope of 1/2 whereas the DP resonator exhibits a slope of 1

Figure 4a shows the frequency response of the PT-symmetric resonator biased initially near the EPs as a function of perturbation. Figure 4b shows the dependence of the extracted frequency splitting on the perturbation strength. For comparison, the frequency response of the resonator operated at the DP was also collected by moving external feedback away. Overall, the frequency splitting of the EP resonator is larger than that of the DP resonator subject to the same small perturbations, as expected. For $\delta =4 \%$, as shown in Fig. 4b, an enhancement of approximately 5 times is experimentally observed. This shows that the experimental results align well with the theoretical expectations and simulations. Moreover, the sensitivity can be enhanced by an order of magnitude compared to that of the DP resonator for sufficiently small perturbations. The inset in Fig. 4b displays a logarithmic plot of the dependence of Δ*ω*_EP_ and Δ*ω*_DP_ on *δ*. The DP resonator exhibits a slope of 1, whereas the EP resonator exhibits a slope of 1/2, confirming the square-root topology of EPs.

**Fig. 5:**
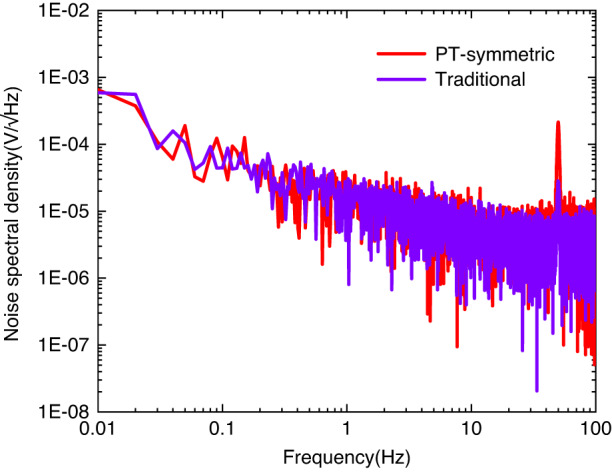
Noise spectral density extracted from the readout channel. Note that the peak in the figure corresponds to 50 Hz power source frequency

Although our PT-symmetric resonators with loss and gain elements have high sensitivity to small perturbations when biased at EPs, the loss and gain elements are prone to adding noise to the system. This issue has raised an ongoing debate concerning the effectiveness of EP sensing schemes^26–28^. There is technical noise and fundamental noise in PT-symmetric systems. Technical noise refers to thermal noise and electronic noise. Fundamental noise refers to the excess noise caused by the eigenbasis collapse in non-Hermitian systems. In the classic system, technical noise is more common. The total root mean square (RMS) noise voltage, *v*_*PT*_, can be expressed as a sum of various terms associated with different noise sources that might affect the precision of the measurements:

5 $${v}_{{PT}}^{2}={v}_{t}^{2}+{v}_{f}^{2}+{v}_{{DC}}^{2}$$

where *v*_*t*_, *v*_*f*_, and *v*_*DC*_ are the thermal RMS noise voltage of mechanical resonators, the electronic RMS noise voltage of gain resonators due to external feedback circuits, and the electronic RMS noise voltage due to bias voltage sources, respectively. Typically, *v*_*DC*_ is dominated by the other terms in the equation. To characterize the noise level of micromechanical resonators, the noise power spectral density (PSD) was measured for traditional and PT-symmetric schemes. During the measurements, the driving signal was turned off, and a noise analyzer (Zurich Instruments HF2LI) was used to measure the noise PSD around the EPs at the readout channel. As shown in Fig. 5, the average values of the noise voltage spectral density for the traditional and PT-symmetric schemes are 0.69 × 10^−5^ $\text{V}/\sqrt{\text{Hz}}$ and 1.05 × 10^−5^ $\text{V}/\sqrt{\text{Hz}}$, respectively. The measured noise spectral density of the EP sensing scheme is slightly greater than that of the traditional scheme. This shows that the noise voltage of the gain resonators due to external feedback circuits is dominant. Noise limits the minimum signal that the sensors can detect; however, *v*_*t*_ and *v*_*f*_ do not experience strong variations around EPs, while the sensitivity is enhanced. As a result, the overall signal-to-noise ratio is still greatly enhanced, which is desirable for various sensors^2^.

Higher sensitivity can potentially be achieved by reducing the noise of the external proportional feedback control circuits, which is currently dominated by circuit parasitics. Detuning of coupled DETF resonators due to process tolerance induces a baseline bifurcation that limits the smallest Δ*ω* that can be detected. This corresponds to zero outputs in general sensors.

Resonators for resonant sensors are usually made to be as lossless as possible to exhibit high quality factors^9^, or the effective quality factor of the resonators is further improved by external proportional feedback control^35^. For our demonstration, we have utilized the same configurations, including implementing the same closed-loop feedback design as those in mode-localized resonators^8,9^. In principle, hence, the EP resonator presented here does not bring any additional noise relative to the mode-localized resonators. However, shifts in amplitude may not be as accurately measured as those in frequency. EPs also exist in coupled resonators with unbalanced gain and loss^14,15^. Such unbalanced systems could be exploited to enhance the sensitivity of high-loss resonators. Previous EP-based sensors in which the perturbation is exerted on one of the coupled resonators cause PT symmetry to break during operation, leading to complex frequency splitting^25^. The perturbation in our scheme is exerted on the coupling spring, which is symmetric about the two coupled resonators, leading to real frequency splitting. However, the proportional coefficient in the symmetric perturbation is not as large as that in the asymmetric perturbation^32^. The application of EP-based silicon micromechanical resonators as well as their noise properties remains an important direction for future work.

## Conclusions

In summary, we present both theoretical and experimental studies of a PT-symmetric micromechanical resonator. We show that the frequency splitting induced by a perturbation at an exceptional point has a square-root dependence on the perturbation strength, in contrast to the linear dependence in traditional resonators, leading to enhanced sensitivity for small perturbations. Simulations and measurements from a pair of mechanically coupled micromechanical resonators support the theoretical predictions. By replacing the perturbation with acceleration or magnetic signals, our scheme may find applications in accelerometers and magnetometers.

## Methods

### Coupled-mode equations for micromechanical resonators

Applying Newton’s law to coupled micromechanical resonators in Fig. 1b yields the following equations:

6 $$\begin{array}{c}m{\ddot{x}}_{1}-c{\dot{x}}_{1}+k{x}_{1}+{k}_{c}\left({x}_{1}-{x}_{2}\right)=0\\ m{\ddot{x}}_{2}+c{\dot{x}}_{2}+k{x}_{2}+{k}_{c}({x}_{2}-{x}_{1})=0\end{array}$$

where *m*, *k*, and *c* are the mass, spring constant, and damping coefficient of a single resonator, respectively, and *k*_*c*_ is the coupling spring constant. *x*_*n*_ (n = 1,2) denotes the vibration displacements of the two resonators. Taking ${x}_{{\rm{n}}}\left({\rm{t}}\right)\to {x}_{{\rm{n}}}{e}^{i\omega t}$, we rewrite Eq. (6) as

7 $$\left[\begin{array}{cc}1+\mu -i\omega g-{\omega }^{2} & -\mu \\ -\mu & 1+\mu +i\omega \gamma -{\omega }^{2}\end{array}\right]\left[\begin{array}{c}{x}_{1}\\ {x}_{2}\end{array}\right]=0$$

where $\mu ={k}_{c}/k$ is the coupling strength,$\gamma =c/\sqrt{{mk}}$ is the loss strength, $g=c/\sqrt{{mk}}$ is the gain strength, and $\omega$ is scaled by ${\omega }_{0}=\sqrt{k/m}$.

If the coupling is weak, i.e., $\mu \ll 1$, $\mu$ and $\gamma$ can be taken as the same order, and we make the following approximations: ${\omega }^{2}\approx 2\omega -1$, $\omega \gamma \approx \gamma$, and $\omega g\approx g$. Equation (7) is then reduced to

8 $$\left[\begin{array}{cc}1+\frac{\mu -{ig}}{2}-\omega & -\frac{\mu }{2}\\ -\frac{\mu }{2} & 1+\frac{\mu +i\gamma }{2}-\omega \end{array}\right]\left[\begin{array}{c}{x}_{1}\\ {x}_{2}\end{array}\right]=0$$

Equation (8) is equivalent to the coupled-mode equations in Eq. (2) with time-harmonic displacement ${x}_{{\rm{n}}}\left({\rm{t}}\right)\to {x}_{{\rm{n}}}{e}^{i\omega t}$. Solving Eq. (8) yields

9 $${\omega }_{\pm }=1+\frac{\mu }{2}+i\frac{-g+\gamma }{4}\mp \frac{1}{2}\sqrt{{\mu }^{2}-{\left(\frac{g+\gamma }{2}\right)}^{2}}$$

When under a delicate balance between gain and loss $g=\gamma$, i.e., the PT-symmetric dimer, the eigenfrequencies and the corresponding eigenstates are defined by Equation (4).

For the PT-symmetric dimer, the frequency splitting near EPs is described by

10 $$\triangle {\omega }_{{\rm{EP}}}={\omega }_{+}-{\omega }_{-}=\sqrt{{\mu }^{2}-{\gamma }^{2}}$$

When the coupling spring is subjected to an external perturbation, the coupling spring constant ${k}_{c}$ is altered to ${k}_{c}+\triangle {k}_{c}$, corresponding to the coupling strength $(1+\delta )\mu$, where $\delta =\triangle {k}_{c}/{k}_{c}$. The frequency splitting at EPs ($\mu =\gamma$) due to the perturbation is given by

11 $$\triangle {\omega }_{{\rm{EP}}}={\omega }_{+}-{\omega }_{-}=\sqrt{{\left(1+\delta \right)}^{2}{\mu }^{2}-{\gamma }^{2}}\approx \mu \sqrt{2\delta }$$

The sensitivity of the frequency splitting to perturbation is given by

12 $${S}_{{\rm{EP}}}=\frac{\partial \triangle {\omega }_{{EP}}}{\partial \delta }\approx \frac{\mu }{\sqrt{2\delta }}$$

Taking $g=\gamma =0$ in Eq. (8), we obtain the Hermitian Hamiltonians

13 $$\left[\begin{array}{cc}1+\frac{\mu }{2}-\omega & -\frac{\mu }{2}\\ -\frac{\mu }{2} & 1+\frac{\mu }{2}-\omega \end{array}\right]\left[\begin{array}{c}{x}_{1}\\ {x}_{2}\end{array}\right]=0$$

The characteristic equation is then expressed as

14 $${\left(1+\frac{\mu }{2}-\omega \right)}^{2}-{\left(\frac{\mu }{2}\right)}^{2}=0$$

Solving the characteristic equation yields

15 $${\omega }_{\pm }=1+\frac{\mu }{2}\pm \frac{\mu }{2}$$

For the Hermitian system, the frequency splitting near DPs is described by

16 $$\triangle {\omega }_{{\rm{DP}}}={\omega }_{+}-{\omega }_{-}=\mu$$

The DPs appear when $\mu =0$. Hence, the traditional resonators become trivially degenerate when uncoupled from each other, $\mu =0$. Under coupling and subject to the perturbation *δ*, the resulting frequency splitting at the DPs is given by

17 $$\triangle {\omega }_{{\rm{DP}}}=\mu \delta .$$

### Fabrication of silicon micromechanical resonators

Silicon micromechanical resonators were fabricated using n-type (100) silicon-on-insulator (SOI) wafers. The process flow is presented in Supplementary Fig. 4. Each of the tines in the tuning-fork resonators was designed to be 20 μm thick, 300 μm long, and 8 μm wide, with a gap of 6 μm between the tines. The drive and coupling gaps were designed to be 3 μm wide.

### Gain resonators

A gain resonator was achieved by applying a feedback force proportional to its velocity $\dot{{\rm{x}}}$. This force is expressed as

18 $${{\rm{F}}}_{{\rm{v}}}={{\rm{c}}}_{{\rm{e}}}\dot{{\rm{x}}}$$

Under the feedback force, the dynamic equation is given by

19 $${\rm{m}}\ddot{{\rm{x}}}+{\rm{c}}\dot{{\rm{x}}}+{\rm{kx}}={{\rm{F}}}_{{\rm{v}}}$$

Note that due to the presence of the feedback force, the effective damping coefficient of the resonator is modified into

20 $${{\rm{c}}}_{{\rm{eff}}}={\rm{c}}-{{\rm{c}}}_{{\rm{e}}}$$

Therefore, the damping coefficient can be adjusted by regulating the feedback force (Supplementary). In the vibration equation, a positive damping coefficient represents a loss, and a negative damping coefficient represents a gain.

### A perturbation approach

Weak coupling between two silicon micromechanical resonators was achieved by using a flexure beam. The flexure beam was designed with a long beam 50 μm long and 5 μm wide and a short beam 25 μm long and 5 μm wide. Two DETFs were weakly coupled by a flexure beam connected to their ends. The initial coupling coefficient k_c_/k was 0.00285. The counter electrode was designed in a gap of 3 μm relative to the flexure beam. The equivalent spring stiffness ${{\rm{k}}}_{{\rm{eff}}}$ of the flexure beam can be electrostatically adjusted. ${{\rm{k}}}_{{\rm{eff}}}$ is expressed as

21 $${{\rm{k}}}_{{\rm{eff}}}={{\rm{k}}}_{{\rm{c}}}+{{\rm{k}}}_{{\rm{e}}}={{\rm{k}}}_{{\rm{c}}}+\triangle {{\rm{k}}}_{{\rm{c}}}=(1+\delta )\mu$$

where ${{\rm{k}}}_{{\rm{c}}}$ and ${{\rm{k}}}_{{\rm{e}}}=\triangle {{\rm{k}}}_{{\rm{c}}}$ are the mechanical-spring stiffness and the electrical spring stiffness, respectively, and a perturbation ${\rm{\delta }}=\triangle {{\rm{k}}}_{{\rm{c}}}/{{\rm{k}}}_{{\rm{c}}}$. By changing the voltage across the counter electrode and the flexure beam, the perturbation can be adjusted (Supplementary Fig. 3c).

### Measurement setup

Micromechanical resonators were placed in a customized vacuum chamber. The gain resonator was controlled and sensed using parallel-plate capacitive transduction. In the feedback circuit of the gain resonator, a transimpedance amplifier (TIA) (OPA656) was used to convert the motion signal of the resonator into an electrical signal. A bandpass filter (BPF) was used to prevent the possible occurrence of unwanted oscillator modes. The voltage control amplifier (VCA810) was used for the voltage amplitude control, and the subsequent electrical signal flowed to the phase modulation, enabling the phase to be consistent with the movement velocity of the resonator. The final output electrical signal was used as the driving signal of the resonator together with the AC source of 20 mVpp. The gain resonator was biased by a DC voltage of 25 V. The feedback circuit was powered by +5/−5 V DC voltage. The loss resonator with parallel-plate capacitive transduction was used only for measurement. The frequency response was recorded using a lock-in amplifier (HF2LI, Zurich Instruments) connected to the loss resonator. The gain resonator and the loss resonator were both connected to GND. A photograph of the experimental setup is provided as Supplementary Fig. 5b.

### Supplementary information

Supplementary Information

## Data Availability

The data that support the plots within this paper and other findings of this study are available from the corresponding author upon reasonable request.
