# Supplementary material for: Exceptional points enhance sensing in silicon micromechanical resonators
Source: Microsyst Nanoeng. 2024 Jan 19;10:12. doi: 10.1038/s41378-023-00641-w (PMC10796675; doi:10.1038/s41378-023-00641-w)
Supplement: Supplementary file 1 — Supplementary Information [file 41378_2023_641_MOESM1_ESM.docx]

**Supplementary**

**Exceptional points enhance sensing in silicon micromechanical resonators**

__________________________________________________________________________________________________________________

**Man-Na Zhang^1^, Lei Dong^1^, Li-Feng Wang^1*^ & Qing-An Huang^1*^**

1 Key Laboratory of MEMS of the Ministry of Education, Southeast University, Nanjing 210096, China.

*e-mail: wanglifeng@seu.edu.cn; hqa@seu.edu.cn

A. Simulations of PT-symmetric silicon micromechanical resonators

*A.1****. Lumped Mechanical System interface***

In order to verify the exceptional point enhanced sensing in silicon micromechanical resonator, we first use the Lumped Mechanical System interface in COMSOL Multiphysics^1^. The schematic diagram of the model is shown in Supplementary Fig. 1a and the corresponding configuration is shown in Supplementary Fig. 1b. The initial setting details of parameters for PT symmetric resonators are as follows:

Spring constant $k_{1}=k_{2}=1000 N/m$, coupling spring constant $k_{c}=10 N/m$, mass $m_{1}=m_{2}=4e^{-9}kg$, damping coefficient $c_{1}={-c}_{2}=-2e^{-5}N\cdot s/m$.

Under this setting, for the case of the resonator operating at EPs, the gain/loss coefficient $g=\gamma={c_{1}}/\sqrt{mk}=0.01$, resonant frequency $\omega_{0}=\sqrt{k/m}=5e^{5} rad/s$ and initial coupling coefficient can be obtained by $\mu={k_{c}}/{k_{1}}=0.01$. For traditional coupling resonators operating at DPs, the loss coefficient $\gamma$ of the DP resonators should be 0 in the strict sense, but in practice it is usually considered to be very small. Therefore, the damping coefficient is chosen here $c_{1}=c_{2}=-2e^{-6}N\cdot s/m$ while the spring constant $k_{1,2}$ and mass $m_{1,2}$ are the same as above. In this case, the loss coefficient is $\gamma={c_{1}}/\sqrt{mk}=0.001$ and the resonant frequency is $\omega_{0}=\sqrt{k/m}=5e^{5} rad/s$ in the traditional coupling resonator. It should be noted that the DP resonator requires the coupling coefficient to be equal to 0, so the initial value of coupling spring constant $k_{c}$ is 0.

Fig.1*c* and *d* in the article show the simulation results.

**Supplementary Fig.1.** **a,** Schematic diagram of Lumped Mechanical Model of micromechanical resonators. **b,** The configuration window of the Lumped Mechanical System (LMS) interface in COMSOL Multiphysics.

*A.2****. Solid Mechanics interface***

In order to further explore the characteristics of PT-symmetric micromechanical resonators, we have established a complete finite element model of the mechanically coupled micromechanical resonator by using solid mechanics interface in COMSOL Multiphysics^1^. As seen in Supplementary Fig. 2a, the gain and loss elements of the PT- symmetric resonators are modeled by adding the opposite imaginary part^2^ represented by the gain coefficient $g$ and the loss coefficient $\gamma$ to Young's modulus. The mechanically coupled beam is modeled as a neutral element without gain and loss. The corresponding computational grid of the finite element simulation is shown in Supplementary Fig. 2b, and the parameters used in the simulation are listed in Supplementary Table 1. Here for the convenience of analysis, we consider a special case for $g=\gamma$. Also, the phase between resonator 1 and resonator 2 is also plotted.

**Supplementary Table 1.** Parameters used in finite element simulation.

| **Parameter** | **Value** |
| --- | --- |
| Beam Length | 300 μm |
| Beam Width | 8 μm |
| Electrode Gaps | 3 μm |
| Device Thickness | 20 μm |
| Coupling Long Beam (×4) | 50×5 μm |
| Coupling Short Beam (×3) | 25×5 μm |
| Young's modulus | 150 Gpa |
| Poisson's ratio | 0.27 |
| Density | 2350 kg/m^3^ |


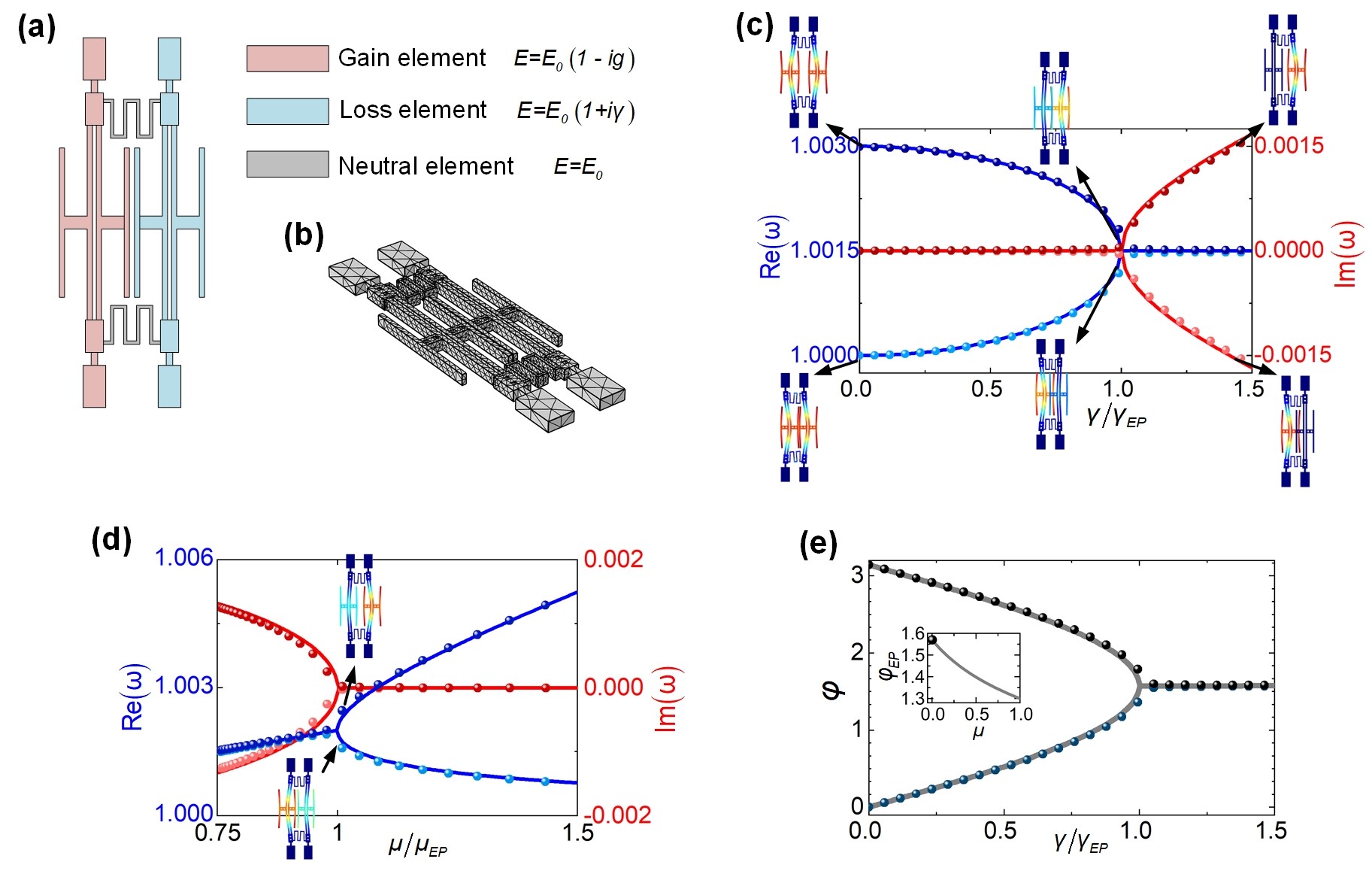


**Supplementary Fig. 2. a**, Schematic diagram of the finite element simulation domain of the micromechanical resonator. **b,** Computational grid for finite element simulation in COMSOL Multiphysics. **c,** Parameter evolution of the real and imaginary parts of the eigenfrequency, versus the gain/loss coefficient $\gamma$. Here, the coefficient $\mu$ is taken as 0.003. **d,** Parameter evolution of the real and imaginary parts of the eigenfrequency, versus the coupling coefficient $\mu$. Here, gain/loss coefficient $g=\gamma=$0.004. **e,** Parameter evolution of $\varphi$, versus the gain/loss coefficient $\gamma$. The inset shows the variation of $\varphi_{PT}$ with the coupling coefficient $\mu$ at EP. The line is the theoretical results and the symbol is the simulation results.

***A.3. Simulation of perturbations***

In order to introduce perturbations to the coupling coefficient of the mechanically coupled resonator, we have designed a mechanically coupled beam as shown in Supplementary Fig. 3a. The mechanical coupling beam is connected at the two ends of the resonator, the counter electrode and the flexure mechanical beam are directly placed opposite with each other in a gap of 3 μm. When we apply a voltage across the two electrode, the perturbation $\delta$ is introduced to the coupling coefficient^3^. The two perturbation electrodes are simultaneously connected to an external voltage, thereby the coupling coefficient increases by $\mu=\left( 1+\delta\right)\mu_{0}$ where $\mu_{0}$ denotes the initial coupling coefficient. The coupling coefficient $\mu$ can be calculated by low frequency $\omega_{-}$ and high frequency $\omega_{+}$ in the frequency response of the coupled resonator:

$\mu=\frac{1}{2}\frac{\omega_{+}^{2}-\omega_{-}^{2}}{\omega_{-}^{2}}$ (S1)

For the designed perturbation structure, the flexure beam consists of a long beam with a size of 50 μm×5 μm and a short beam with a size of 25 μm×5 μm. We use Solid Mechanics interface and Electrostatics interface in COMSOL Multiphysics to simulate the coupling coefficient as a function of applied voltage. The finite element simulation grid is shown in Supplementary Fig. 3b. Supplementary Fig. 3c plots the relationship between the applied voltage and the perturbation $\delta$. The dots represent the finite element simulation results and the line represents the theoretical results. In the upper illustration, gray areas are used for drawing the perturbation used in our experiments. The ordinate is the frequency splitting corresponding to the perturbation $\delta$.


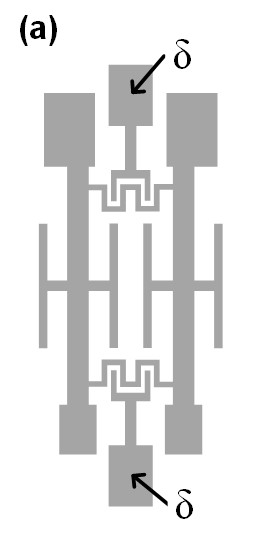

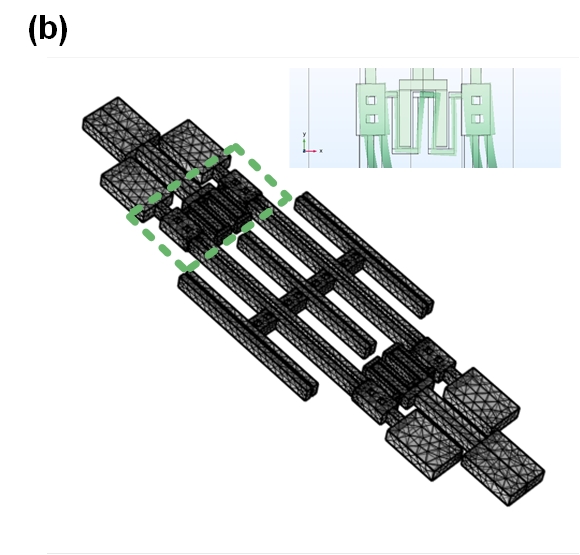

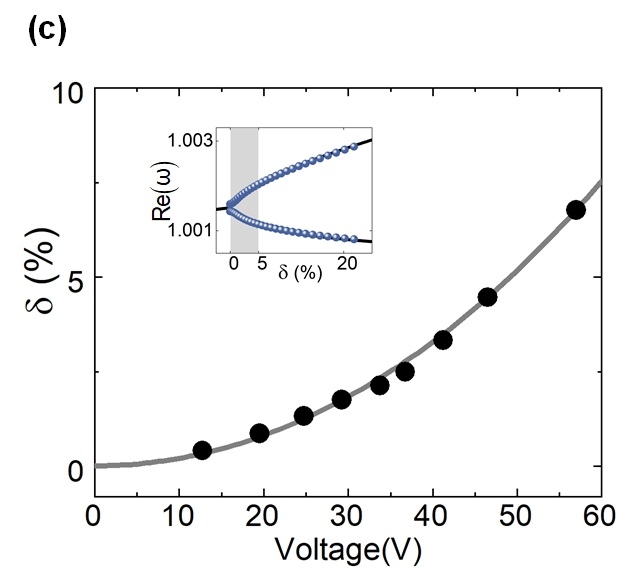


**Supplementary Fig.3.** **a,** The structures designed for mechanically coupled resonators to introduce perturbations to the coupling coefficient. **b,** Computational grid for finite element simulation in COMSOL Multiphysics. **c,** The relationship between the applied voltage and the perturbation $\delta$. The symbols in black are experimental results. The upper illustration is the perturbation region of the experiments in this paper, where the gray symbols are the simulated results.

B. Fabrication of silicon micromechanical resonators

The silicon micromechanical resonator used in the experiment was fabricated on n-type SOI wafers with <100> crystal orientation. The specific process is as follows (Supplementary Figure 4):

1. Prepare SOI wafers with the device layer thickness of 20 μm, and clean the SOI wafers using the RCA standard cleaning method to remove surface contaminants.
2. The deposited phosphosilicate glass layer (PSG) on the device layer was doped by a high-temperature diffusion process to reduce its resistivity to less than 0.02Ω·cm, so that it formed a good ohmic contact with the aluminum electrode.
3. A 100/200 nm layer Mo/Au metal was sputtered on the surface of the device layer and then patterned as an electrode. The uniformity of the metal film thickness is over ±4%, and the deposition rate is about 47.8 nm/min.
4. Etching the structure layer by deep reactive ion etching (DRIE) process with etching gas SF_6_ and passivation gas C_4_F_8_, the etching stopped when it reached the SiO_2_ layer, so as to obtain the designed structure of the resonators.
5. Gaseous HF acid was used to release the oxide layer, and the release process was divided into two steps. The step one is vertical etching of exposed oxide with etching rate of about 0.05 μm/min; the step two is lateral etching of undercut oxide with etching rate of about 0.1 μm/min. This step forms a cavity between the substrate and the device layer, releasing the vibrating structure of the resonator.
6. It shows the scanning electron micrograph of the resonator after the processing, where the main body is a tuning fork with 300 μm long, 8 μm wide, and a spacing of 6 μm.
7. The resonator was pasted on a customized circuit board with high temperature resistant glue, and we use aluminum wires to connect the electrodes of the resonator to the interface of the circuit board to complete the simple board connections of the resonator.


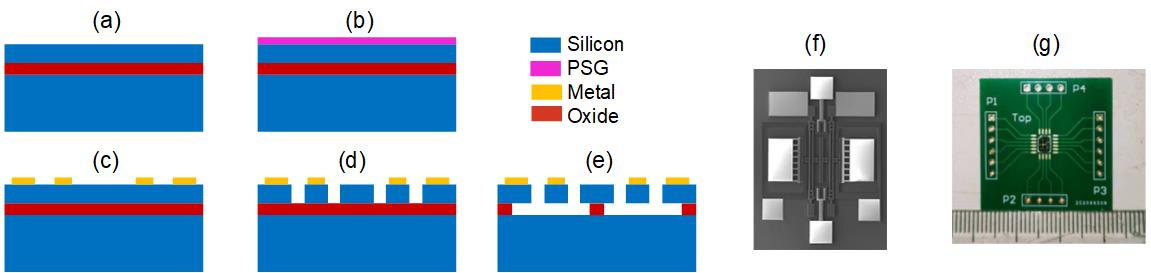


**Supplementary Fig. 4.** SOI manufacturing process.

C. Implementation of gain resonators

In our experiment, the gain resonator was realized by an external feedback control circuit, including trans-impedance amplifier (TIA), band pass filter (BPF), gain control module, and phase modulation module. In the process of external feedback, the motional current of the resonator $i_{sense}$ flows into the TIA and converts it into an electrical signal, which is described by^4^:

$i_{sense}=\frac{\partial Q}{\partial t}=V\frac{\partial C}{\partial t}=V\frac{\partial C}{\partial x}\frac{\partial x}{\partial t}=V\left( \frac{\varepsilon A}{d_{0}^{2}}-\frac{2\varepsilon A}{d_{0}^{3}}x+\frac{3\varepsilon A}{d_{0}^{4}}x^{2}-\frac{4\varepsilon A}{d_{0}^{5}}x^{3}-\ldots\right)\dot{x}\approx V\frac{\varepsilon A}{d_{0}^{2}}\dot{x}$ (s2)

where $C$ represents the capacitance between the sensing electrode plates, $V$ represents the voltage difference between the sensing electrode plates, $A$ represents the area of the sensing electrode plates, $d_{0}$ represents the initial spacing of the sensing electrode plates, $\varepsilon$ represents the dielectric constant of the environment, and $x$ represents the displacement of the resonator. There is a non-linear term related to the amplitude $x$ in the above formula, which can be ignored when the resonator is in a small deformation. Therefore, the magnitude of the detected current signal $i_{sense}$ is approximately proportional to the velocity of resonator.

After the detected current signal is filtered by BPF, and further corrected by the gain control module and the phase modulation module, the feedback loop obtains an electrical signal that is completely related to the velocity of the resonator, namely $V_{fb}=A_{fb}\dot{x}$, where $V_{fb}$ is the voltage signal output by the feedback loop, and $A_{fb}$ is the overall gain of the feedback loop. The voltage signal $V_{fb}$ is fed back to the drive electrode of the resonator through electrostatic force. For a drive electrode with a DC bias voltage $V_{dc}$ of between the plates, the overall feedback force can be written as:

$F_{v}=\frac{1}{2}\frac{\partial C}{\partial d}\left( V_{dc}+V_{fb} \right)^{2}=\frac{1}{2}\frac{\partial C}{\partial d}\left( V_{dc}+A_{fb}\dot{x} \right)^{2}\approx\frac{1}{2}\frac{\varepsilon A}{d_{0}^{2}}\left( V_{dc}^{2}+2V_{dc}A_{fb}\dot{x}+A_{fb}^{2}\dot{x}^{2} \right)$ (s3)

It can be seen that the first term in Eq. (s3) is a constant term that can only adjust the equilibrium point of the resonator, the second term is the useful term that we expect to adjust the damping proportional to the velocity of resonator, and the third term is high-frequency components that can be ignored. In this case, the feedback electrostatic force can be considered as^4^

$F_{v}=\frac{\varepsilon A}{d_{0}^{2}}V_{dc}A_{fb}\dot{x}=c_{f}\dot{x}$ (s4)

For a resonator with mass $m$, stiffness $k$, and damping coefficient $c$, the vibration equation considering the feedback force can be written as:

$m\ddot{x}+c\dot{x}+kx=F_{v}$ (s5)

Note that due to the presence of the feedback force, the effective damping coefficient of the resonator is now changed into:

$c_{g}=c-c_{f}$ (s6)

Therefore, the equivalent damping of the resonator is adjusted, and the gain resonator can be established in this way. In our experiment, in order to establish the gain resonator in the PT symmetric system, it is necessary to ensure that the value of $c_{g}$ is around $-$4.77×10^-6^ $N\cdot s/m$. Therefore, from the equation (s4) we can get the gain $A_{fb}$ that the feedback circuit needs to set.

D. Measurement set-up

The schematic flow diagram of experiments is shown in Supplementary Fig. 5a, and the corresponding measurement platform of silicon micromechanical resonators is shown in Supplementary Fig. 5b. The tests of the silicon micromechanical resonator in this paper was carried out on the damping isolation platform produced by Newport of United States with a surface of 4.8 mm thick ferromagnetic 430 stainless steel. The resonator was pasted on a customized transfer circuit printed board, and the electrodes of the micromechanical resonator were connected to the interface of the transfer circuit printed board using gold wires. The entire micromechanical resonator was placed in a customized vacuum chamber of which pressure can be adjusted by an automatic pressure controller in the range of 7~7×10^5^ mtorr. The vacuum pump model D16C of Germany Leybold Company was connected to the vacuum chamber through a vacuum bellows model KF25 to extract gas from the chamber.

In the feedback circuit of the gain resonator, OPA656 was used in the transimpedance amplifier (TIA) to convert the motional current of the resonator into an electrical signal. A band pass filter (BPF), which is used to remove the possible occurrence of unwanted oscillator modes, was then connected. The voltage control amplifier VCA810 was used as the main chip for the voltage amplitude limit of the gain control, and the subsequent electrical signal was connected to the phase modulation to correct the phase to be consistent with the movement velocity of the resonator. Then the final electrical signal was used as the driving signal of the resonator together with the AC source of 20 mVpp, and the damping of the resonator was adjusted by the corresponding electrostatic force. The entire feedback circuit is powered by +5/-5V DC voltage.

In the PT symmetric micromechanical resonators, the initial resonance frequency of the loss resonator and gain resonator was both 302.36 kHz. The initial coupling coefficient of 0.00285 was obtained. Usually, additional careful adjustment of the DC bias was required to obtain the initial mechanical symmetry of the two resonators. The frequency response of micromechanical resonators was characterized using a lock-in amplifier HF2LI of Zurich Instruments with the scan frequency range from 302.4 kHz to 303.2 kHz. The resonator was connected to GND and the sensing electrodes was connected to 25V DC voltage. The two perturbation electrodes were connected to the perturbation voltage at the same time. In the feedback loop of the gain resonator, the gain was adjusted to an appropriate value. A transimpedance amplifier was carefully configured to connect to the loss resonator to obtain the frequency response of the system.

**(a)**

**Supplementary Fig.5.** **a,** Schematic flow diagram of the experiments. **b,** Measurement set-up for the silicon micromechanical resonators.

The DP resonator in the traditional coupling system was achieved by carefully removing the gain side of feedback circuit in the EP system, and the electrostatic coupling was used instead of the mechanical coupling to achieve the DP with degenerate eigenfrequencies. To operate the coupled resonator under the DP, the pressure of the vacuum chamber was set to 50 mtorr and the resonant frequencies of the two resonators was set to be infinitely close to each other and remained constant. The corresponding frequency response is shown in Supplementary Fig.6.


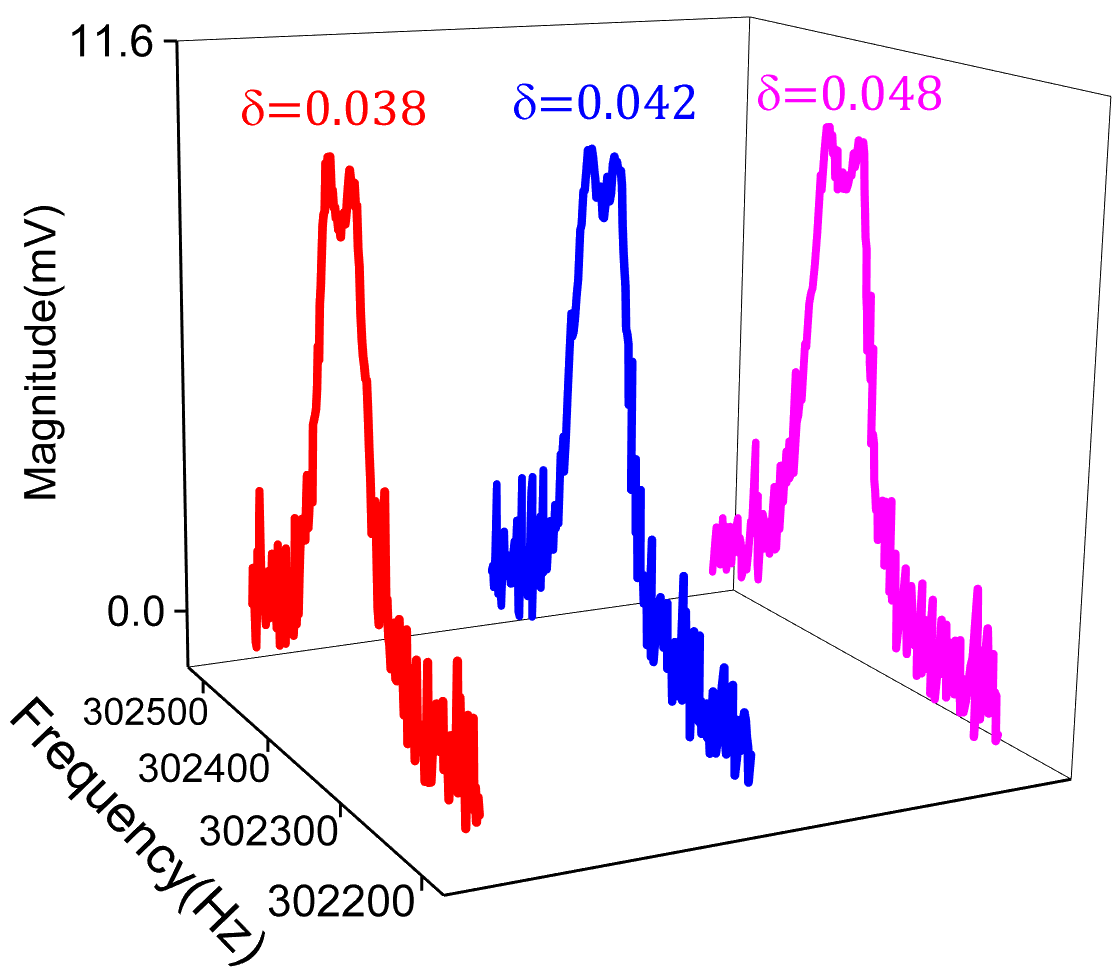


**Supplementary Fig. 6.** Frequency response of DP resonators under a perturbation $\delta$ introduced by the coupling coefficient

**References**

1. COMSOL MULTIPHYSICS®, [www.comsol.com](http://www.comsol.com).
2. Yasumura, K. Y., Stowe, T. D., Chow, E. M., Pfafman, T., Kenny, T. W., Stipe, B. C. and Rugar, D. Quality factors in micron- and submicron-thick cantilevers. *J. Microelectromech. Syst.*, vol. 9, 117-125, 2000.
3. de Laat, M. L. C., Pérez Garza, H. H., Herder, J. L. & Ghatkesar, M. K. A review on in situ stiffness adjustment methods in MEMS. *J. Micromech. Microeng.* Vol.26, 063001, 2016.
4. Sayed, S. and Clark, J. V. Active Control of Effective Mass, Damping and Stiffness of MEMS. *Symp. Des., Test, Integr. Packag. MEMS/MOEMS*, *DTIP*, 6559441, 2013.
